# Patient-Reported Outcomes among Multiple Myeloma Patients Treated with Standard of Care Idecabtagene Vicleucel

**DOI:** 10.3390/cancers15194711

**Published:** 2023-09-25

**Authors:** Laura B. Oswald, Lisa M. Gudenkauf, Xiaoyin Li, Gabriel De Avila, Lauren C. Peres, Kedar Kirtane, Brian D. Gonzalez, Aasha I. Hoogland, Oanh Nguyen, Yvelise Rodriguez, Rachid C. Baz, Kenneth H. Shain, Melissa Alsina, Frederick L. Locke, Ciara Freeman, Omar Castaneda Puglianini, Taiga Nishihori, Hien Liu, Brandon Blue, Ariel Grajales-Cruz, Heather S. L. Jim, Doris K. Hansen

**Affiliations:** 1 Department of Health Outcomes and Behavior, Moffitt Cancer Center, 12902 USF Magnolia Dr., Tampa, FL 33216, USA; lisa.gudenkauf@moffitt.org (L.M.G.); shelly.li@moffitt.org (X.L.); brian.gonzalez@moffitt.org (B.D.G.); aasha.hoogland@moffitt.org (A.I.H.); oanh.nguyen@moffitt.org (O.N.); yvelise.rodriguez@moffitt.org (Y.R.); heather.jim@moffitt.org (H.S.L.J.); 2 Department of Blood and Marrow Transplant and Cellular Immunotherapy, Moffitt Cancer Center, 12902 USF Magnolia Dr., Tampa, FL 33216, USA; gabriel.deavila@moffitt.org (G.D.A.); melissa.alsina@moffitt.org (M.A.); frederick.locke@moffitt.org (F.L.L.); ciara.freeman@moffitt.org (C.F.); omar.castanedapuglianini@moffitt.org (O.C.P.); taiga.nishihori@moffitt.org (T.N.); hien.liu@moffitt.org (H.L.); doris.hansen@moffitt.org (D.K.H.); 3 Department of Cancer Epidemiology, Moffitt Cancer Center, 12902 USF Magnolia Dr., Tampa, FL 33216, USA; lauren.peres@moffitt.org; 4 Department of Head and Neck-Endocrine Oncology, Moffitt Cancer Center, 12902 USF Magnolia Dr., Tampa, FL 33216, USA; kedar.kirtane@moffitt.org; 5 Department of Malignant Hematology, Moffitt Cancer Center, 12902 USF Magnolia Dr., Tampa, FL 33216, USA; rachid.baz@moffitt.org (R.C.B.); ken.shain@moffitt.org (K.H.S.); brandon.blue@moffitt.org (B.B.); ariel.grajales-cruz@moffitt.org (A.G.-C.)

**Keywords:** quality of life, patient-reported outcomes, multiple myeloma, CAR T-cell therapy

## Abstract

**Simple Summary:**

In clinical trials, patients treated with idecabtagene vicleucel (ide-cel) chimeric antigen receptor T-cell therapy (CAR T) have reported meaningful improvements in patient-reported outcomes, such as health-related quality of life. To test whether these findings are generalizable to the broader, real-world patient population, this study aimed to prospectively characterize patient-reported outcomes (i.e., health-related quality of life, symptom burden) among patients with relapsed/refractory multiple myeloma treated with ide-cel CAR T in standard of care. Patient-reported outcomes were assessed across 14 timepoints from pre-CAR T infusion through day 90 post-infusion. Patients reported significant and meaningful improvements in health-related quality of life and physical well-being by day 60 after CAR T infusion. Overall, most patients had meaningful improvement or maintenance of patient-reported outcomes collected over time. Findings have implications for treatment decision-making, patient education, and supportive interventions to improve patient outcomes post-CAR T.

**Abstract:**

Idecabtagene vicleucel (ide-cel) was the first FDA-approved chimeric antigen receptor T-cell therapy for relapsed/refractory multiple myeloma (RRMM) patients. This was the first study to evaluate patient-reported outcomes (PROs) among RRMM patients receiving ide-cel in standard of care (SOC). We prospectively assessed health-related quality of life (HRQOL) and symptoms from pre-infusion (baseline) through day (D)90 post-infusion. Baseline PRO associations with patient characteristics, mean PRO changes, and time to stable change were evaluated with *t*-tests, linear mixed-effects models, and Kaplan–Meier analyses, respectively. Within-person change scores and minimally important difference thresholds determined clinical and meaningful significance. Participants (*n* = 42) were a median of 66 years old (range: 43–81). At baseline, extramedullary disease was associated with worse physical well-being (*p* = 0.008), global pain (*p* < 0.001), performance status (*p* = 0.002), and overall symptom burden (*p* < 0.001). Fatigue (*p* < 0.001) and functional well-being (*p* = 0.003) worsened by D7 before returning to baseline levels. Overall HRQOL (*p* = 0.008) and physical well-being (*p* < 0.001) improved by D60. Most participants reported PRO improvement (10–57%) or maintenance (23–69%) by D90. The median time it took to stabile deterioration in functional well-being was 14 days. The median time it took to stabile improvement in physical and emotional well-being was 60 days. Overall, RRMM patients reported improvements or maintenance of HRQOL and symptom burden after SOC ide-cel.

## 1. Introduction

Multiple myeloma is the second most common hematologic malignancy in the United States (US) [1] and is incurable, as most patients develop relapsed or refractory multiple myeloma (RRMM) after initial therapy [2,3,4]. Multiple myeloma negatively affects patients’ health-related quality of life (HRQOL) or overall wellbeing, due in part to common and distressing disease- and treatment-related symptoms that can interfere with physical and social functioning [5,6,7,8]. While first-line treatments may improve these patient-reported outcomes (PROs), subsequent treatments for RRMM are less likely to improve HRQOL and symptom burden [9,10].

In March 2021, idecabtagene vicleucel (ide-cel) became the first FDA-approved chimeric antigen receptor T-cell therapy (CAR T) for RRMM patients [11]. FDA approval was based on the Phase II KarMMa trial that showed a 73% overall response rate (ORR), complete response (CR) or better among 33% of patients, and a median 10.7 months response duration [12]. This is a striking improvement in clinical efficacy relative to prior treatments for similar patients, which a recent study showed had an ORR of approximately 32% [13]. In addition, ide-cel resulted in clinically meaningful improvements in PROs, such as pain, fatigue, physical function, and global HRQOL [14]. Most recently, the Phase III KarMMa-3 randomized controlled trial showed that RRMM patients treated with ide-cel had better overall HRQOL, cognitive function, fatigue, and pain relative to patients treated with standard regimens at 20 months post-treatment [15]. Thus, the introduction of ide-cel into standard of care (SOC) offers RRMM patients renewed hope for durable remission and improved PROs.

With the introduction of any therapy into SOC, a key question is whether it performs as well in the real-world as it does in clinical trials [16,17]. Trials often have stringent eligibility criteria that are not necessarily representative of real-world patients. Trial participants also tend to be younger and have better overall health, which can affect downstream outcomes [18]. Recently, our team evaluated 159 RRMM patients treated with SOC ide-cel across 11 institutions in the US Multiple Myeloma Immunotherapy Consortium. Clinical outcomes in SOC were comparable to those reported in KarMMa (e.g., 84% ORR, 42% CR or better, and a median 8.6 months response duration), despite 75% of real-world patients not meeting KarMMa eligibility criteria [19]. Building on this work, this subsequent study aimed to prospectively characterize PROs among real-world RRMM patients treated with SOC ide-cel at a single institution. Consistent with the KarMMa trial, we hypothesized that RRMM patients treated with SOC ide-cel would report improvements in PROs.

## 2. Materials & Methods

### 2.1. Participants and Procedures

PRO data were pooled across two observational studies at Moffitt Cancer Center (Moffitt), an NCI-designated comprehensive cancer center in Tampa, FL. PROs were combined with clinical and outcomes data collected in a retrospective electronic medical record (EMR) review study. Each protocol was approved by the Advarra Institutional Review Board (Pro00046848) or deemed exempt from IRB oversight due to minimal risk (Pro00055609, Pro00044602) and was conducted in accordance with Helsinki Declaration ethical standards. Data are available from the corresponding author upon reasonable request. Participants were adults (≥18 years old) with RRMM, scheduled to receive SOC ide-cel, able to speak and read English, without documented or observable psychiatric or neurologic diagnoses that could preclude participation (e.g., dementia), and able to provide informed consent. Between May 2021 and August 2022, trained research coordinators identified potentially eligible patients in collaboration with providers in Moffitt’s Immune Cell Therapy program. Coordinators screened patients’ EMRs for eligibility and approached patients in person or via telephone to introduce the study, confirm eligibility, and solicit informed consent. Participants were asked to complete PRO assessments at 14 timepoints (Supplementary Table S1): baseline (i.e., enrollment, before CAR T infusion), day of CAR T infusion (day (D)0), daily for one week post-infusion (D1–D6), weekly for one month (D7, D14, D21), and monthly for three months (D30, D60, D90). This timeline was informed by recommendations for monitoring PROs after CAR T [20] and our past work, which showed feasibility [21]. Most participants (93%) completed the baseline assessment on or before the day of conditioning chemotherapy. Assessments were completed using REDCap, a HIPAA-compliant and internet-based data capture tool [22].

### 2.2. Measures

#### 2.2.1. Participant Characteristics and Clinical Outcomes

At baseline, participants reported their demographic characteristics (e.g., date of birth, sex, race, ethnicity) and completed the Charlson Comorbidity Index [23,24]. EMR reviews were conducted for baseline clinical characteristics (e.g., diagnosis, treatment history, KarMMa eligibility) and safety and clinical outcomes through D90 (e.g., toxicities, treatment response, date/cause of progression/death). Moffitt physicians assessed cytokine release syndrome (CRS) and neurotoxicity per the American Society for Transplantation and Cellular Therapy criteria [25], hematologic toxicities per the Common Terminology Criteria for Adverse Events (CTCAE) version 5.0 [26], and CAR T response per International Myeloma Working Group criteria [27].

#### 2.2.2. HRQOL

At all timepoints, except D1–D6, participants completed the 27-item Functional Assessment of Cancer Therapy-General (FACT-G), which assesses overall HRQOL and four well-being domains (i.e., physical, functional, emotional, social) [28]. Participants responded to items on a Likert-type scale from 0 to 4. Higher scale scores indicated better HRQOL. We used published thresholds to indicate clinically low individual scores for overall HRQOL (≤62), functional well-being (≤11), physical well-being (≤15), emotional well-being (≤13), and social well-being (≤16) [29]. We also used published thresholds to indicate clinically low average group-level overall HRQOL (≤70), functional well-being (≤14), physical well-being (≤18), emotional well-being (≤15), and social well-being (≤19) [29]. Minimally important differences (MIDs) of ±4 points for overall HRQOL and ±2 points for each well-being domain determined clinically meaningful changes [29,30,31].

On D1–D6, to reduce burden, participants completed the FACT-G7, which includes 7 items from the FACT-G and assesses cancer patients’ top-priority concerns [32]. Participants responded to items on a Likert-type scale from 0 to 4. Higher scale scores indicated better HRQOL. Average scores ≤ 13 indicated low HRQOL, and an MID of ±2 points determined clinically meaningful changes [29]. A FACT-G7 score was derived from the FACT-G on D0 to facilitate statistical analyses.

#### 2.2.3. Symptom Burden

At all timepoints, except D1–D6, participants completed the 31-item PRO Measurement Information System (PROMIS)-29+2 Profile v2.1, which assesses fatigue, pain interference, sleep disturbance, depression, anxiety, physical function, social function (4 items each), cognitive function (2 items), and global pain (1 item) [33,34]. Participants rated their global pain from 0 to 10, and higher scores indicated worse pain. For all other scales, participants responded to items on Likert-type scales from 1 to 5, and standardized T-scores were calculated (normative M = 50, SD = 10). Higher scores were worse for fatigue, pain interference, sleep disturbance, depression, anxiety (55–59 mild, 60–69 moderate, ≥70 severe), and global pain (1–4 mild, 5–6 moderate, ≥7 severe). Lower scores were worse for physical, cognitive, and social function (≤30 severe, 31–40 moderate, 41–45 mild). MIDs of ±2 points for global pain and ±5 points for all other scales determined clinically meaningful changes [35,36]. To minimize confusion between PROMIS physical function and FACT-G physical well-being, we herein refer to PROMIS physical function as “performance status”.

At all timepoints, except D1–D6, participants completed items from the PRO version of the CTCAE (PRO-CTCAE). PRO-CTCAE is a library of 124 items assessing the frequency, severity, and/or interference of 78 toxicities and was designed for investigators to select items that are relevant to specific treatments and/or diagnoses [37,38]. We assessed 31 toxicities (e.g., decreased appetite, nausea, constipation, diarrhea), with 47 items based on consensus among study team experts. Participants responded to items on Likert-type scales from 0 to 4. For each toxicity, we calculated a within-person composite grade that mapped onto clinician-rated CTCAE grades [39], where 0 indicates that a toxicity is absent, 1 = mild, 2 = moderate, 3 = severe, 4 = life-threatening, and 5 = toxicity-related death. Composite grades for patient-reported toxicities were capped at 3 (severe) as recommended [39] and were used to calculate a Toxicity Index to indicate overall symptom burden as follows [40,41]:

$$Toxicity\;Index=\sum_{i=1}^{n}\frac{X_{i}}{\prod_{j=1}^{i-1}\left(1+X_{j}\right)}$$

In this equation, toxicities are ranked in descending order of severity, assigned decreasing weights, and summed. This accommodates the differential impact of multiple toxicities and yields an easily interpretable score, wherein the number before the decimal indicates the highest toxicity grade reported, and the numbers after the decimal indicate other toxicities beyond the highest grade, with lower grade toxicities contributing less to the final score. For example, a participant reporting a single grade 3 toxicity would have a score of 3.0, and a participant reporting one grade 3 and two grade 2 toxicities would have a score of 3.67. We defined the MID threshold for overall symptom burden as a change from one severity category to another (i.e., from ≥3 severe to 2.0–2.9 moderate).

### 2.3. Statistical Analysis

Analyses were performed using SAS Version 9.4. We used descriptive statistics to characterize the sample and summarize the PRO data and independent-sample *t*-tests to evaluate associations between participant characteristics and PROs at baseline. Our analytic approach was informed by the KarMMa trial PRO analyses [14]. First, we used PROC MIXED to calculate linear mixed models, examining changes in average PRO scores from baseline to each follow-up timepoint with maximum likelihood estimation and all available data for calculating estimates. We also used MIDs to determine whether average score changes were clinically meaningful (i.e., exceeded the MID). Second, we used PROC GLIMMIX to calculate logistic regression models, examining differences in the proportion of participants who exceeded clinical thresholds at baseline vs. D90. For HRQOL outcomes, participants were categorized as having low vs. normal HRQOL. For symptom outcomes, participants were categorized as having at least moderate symptoms vs. mild or none. We also used MIDs to quantify the proportion of participants with PRO improvement, maintenance (i.e., no change), or deterioration from baseline to D90. Third, we used Kaplan–Meier analyses to evaluate time to stable PRO change, defined as ≥2 consecutive assessments with clinically meaningful improvement or deterioration [14,42]. Participants who did not achieve stable change by D90 were censored. For all statistical tests, significance was indicated by two-sided *p* < 0.01 to account for multiple comparisons.

## 3. Results

**Figure 1:**
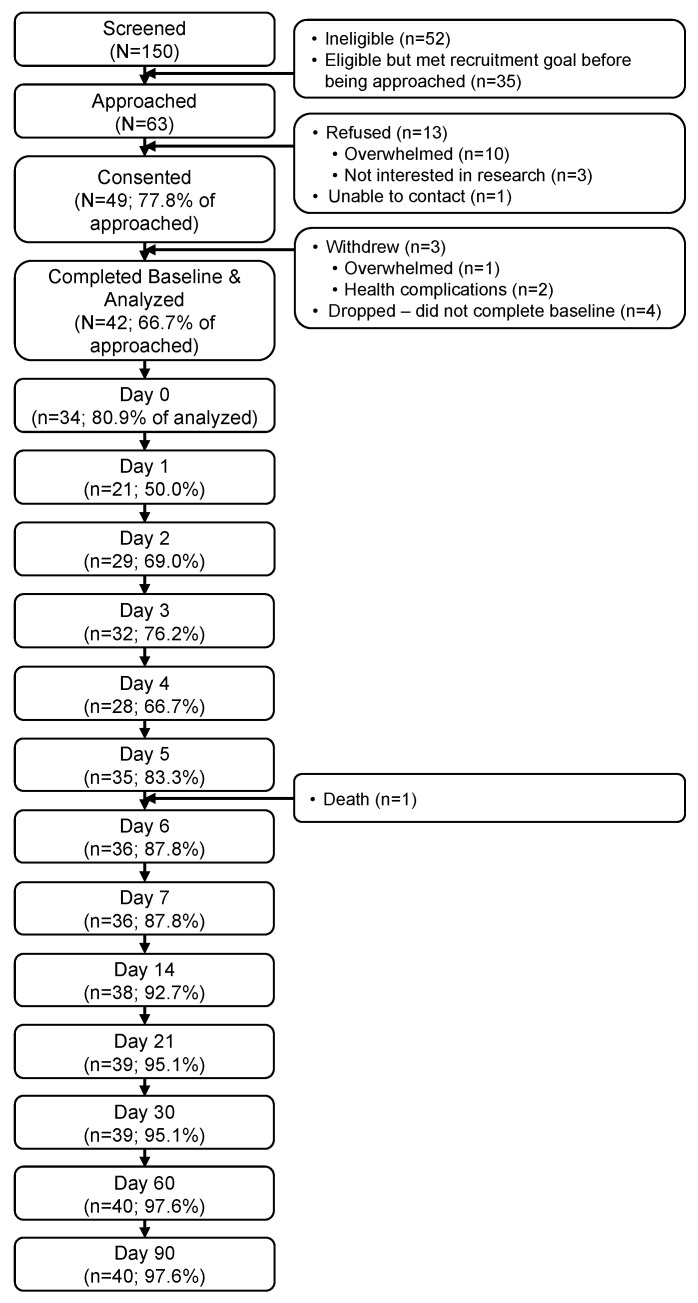
Participant flow through the study.

**Figure 2:**
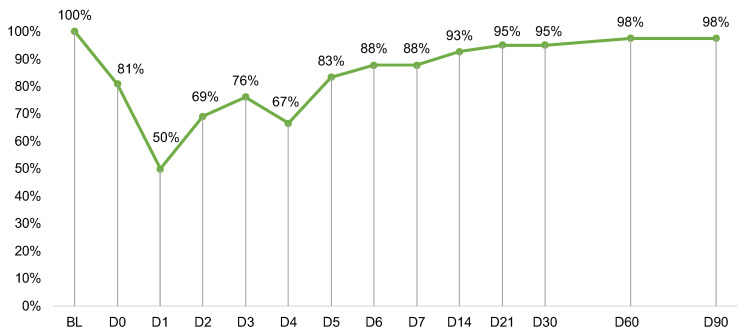
Measure completion rates at each timepoint out of *n* = 42 participants. Day (D)0 was the day of CAR T infusion. Completion rates after D6 were calculated out of *n* = 41 due to one participant death. Abbreviations: BL, baseline.

Sixty-three eligible patients were approached (Figure 1). Of those, 49 (78%) consented to participate, and 42 (67%) provided at least baseline data for analysis. PRO completion rates were lowest during the week post-infusion (D0–D6; range: 50–88%) and high otherwise (D7–D90; range: 88–98%) (Figure 2). Follow-up exploratory analyses compared toxicities between participants who did (*n* = 21) vs. did not complete the D1 assessment (*n* = 21). Participants who did not complete D1 were more likely to develop any grade neurotoxicity (19% vs. 0%) (*p* = 0.035).

### 3.1. Participant Characteristics

As shown in Table 1, participants were a median of 66 years old (range: 43–81), half were male (50%), most identified as non-Hispanic White (69%), and most had earned at least a college degree (57%). The median of prior lines of therapy was 6 (range: 4–16). At least one-third had extramedullary disease (40.5%) and penta-refractory disease (38%). Most (71%) did not meet KarMMa trial eligibility criteria. As shown in Table 2, 38% had a CR or better by D90 post-ide-cel. One participant (2%) died due to disease progression. Most (81%) developed any grade CRS (12% grade ≥ 2), and 10% developed any grade neurotoxicity (5% grade ≥ 2).

### 3.2. Baseline PROs

Table 3 shows average PRO scores over time. At baseline, participants on average reported severe overall symptom burden (M = 3.2, SD = 0.8), mild pain interference (M = 55.0, SD = 10.0), mild global pain (M = 3.7, SD = 2.5), and mildly impaired performance status (M = 41.9, SD = 9.4). At baseline, extramedullary disease was associated with worse physical well-being (*p* = 0.008), worse global pain (*p* < 0.001), worse performance status (*p* = 0.002), and worse overall symptom burden (*p* < 0.001) (Supplementary Table S2). Having achieved at least a college degree was associated with better social well-being (*p* < 0.001).

### 3.3. Mean PRO Changes from Baseline

**Figure 3:**
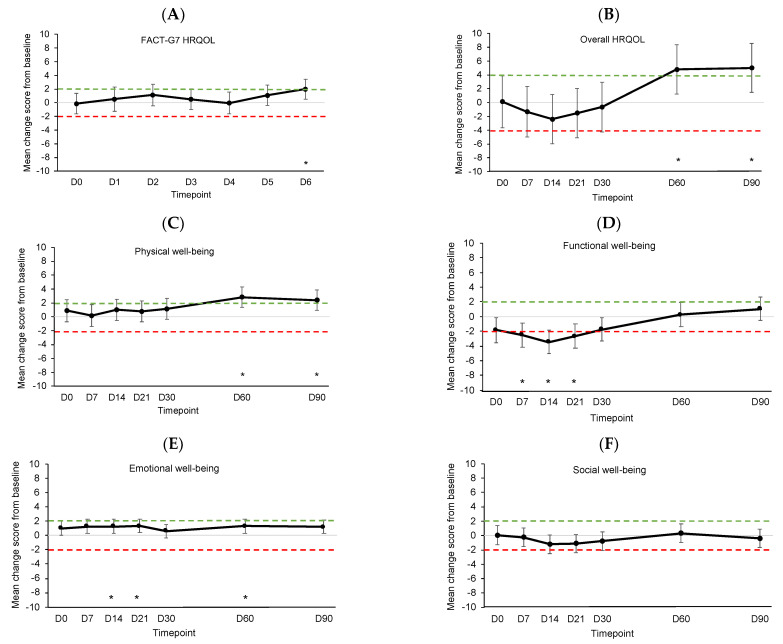
Estimated mean changes from baseline for (**A**) daily HRQOL, (**B**) overall HRQOL, (**C**) physical well-being, (**D**) functional well-being, (**E**) emotional well-being, and (**F**) social well-being. Dotted lines indicate clinically meaningful improvement (green) or worsening (red). Error bars represent 95% confidence intervals. D, day. D0 was the day of CAR T-cell infusion. * *p* < 0.01.

Figure 3 shows the estimated mean changes from baseline for HRQOL outcomes compared to MIDs. See Supplementary Table S3 for details on the linear mixed models. In the week post-infusion, daily HRQOL significantly and meaningfully improved on D6 (*p* = 0.008, Figure 3A). Overall HRQOL and physical well-being significantly and meaningfully improved on D60 (*p* = 0.008, Figure 3B and *p* < 0.001, Figure 3C, respectively), and improvements were sustained at D90 (*p* = 0.006 and *p* = 0.002, respectively). Functional well-being significantly and meaningfully worsened on D7 (*p* = 0.003), D14 (*p* < 0.001), and D21 (*p* = 0.001) before returning to baseline levels (Figure 3D). Emotional well-being showed significant but not meaningful improvements on D14 (*p* = 0.009), D21 (*p* = 0.007), and D60 (*p* = 0.008) (Figure 3E). Social well-being did not change significantly or meaningfully (Figure 3F).

**Figure 4:**
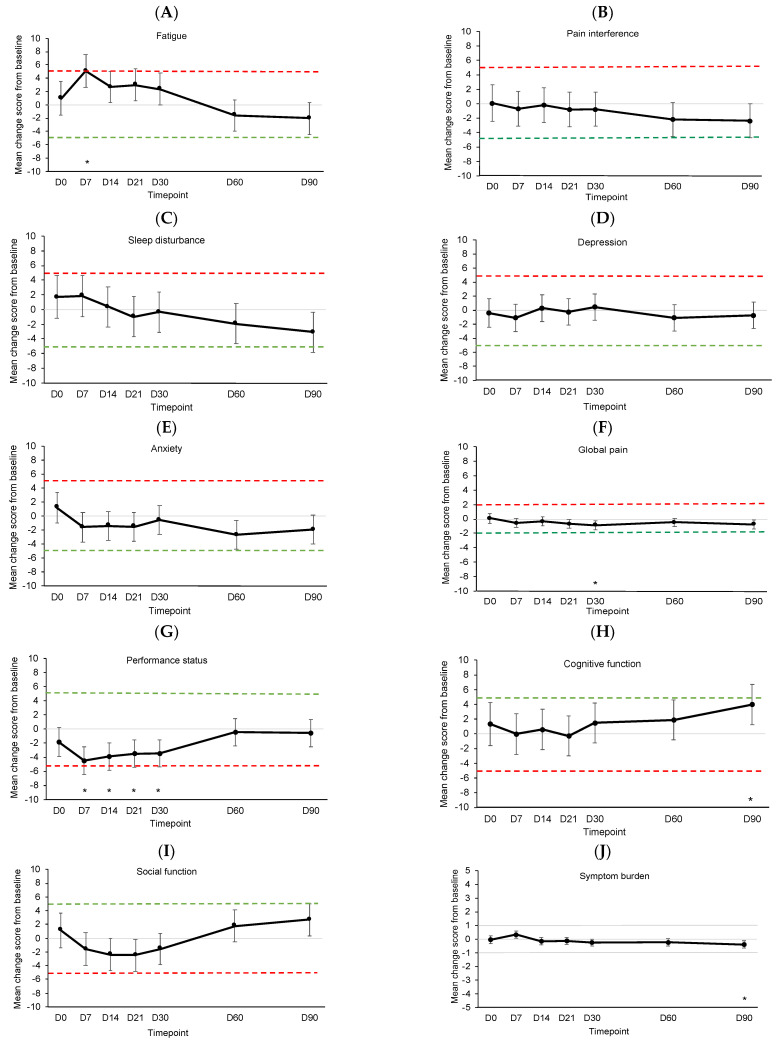
Estimated mean change from baseline for (**A**) fatigue, (**B**) pain interference, (**C**) sleep disturbance, (**D**) depression, (**E**) anxiety, (**F**) global pain, (**G**) performance status, (**H**) cognitive function, (**I**) social function, and (**J**) overall symptom burden. Dotted lines indicate clinically meaningful improvement (green) or worsening (red). Error bars represent 95% confidence intervals. D, day. D0 was the day of CAR T-cell infusion. * *p* < 0.01.

Figure 4 shows the estimated mean changes from baseline for symptom outcomes compared to MIDs. See Supplementary Table S4 for details of the linear mixed models. Fatigue significantly and meaningfully worsened from baseline on D7 (*p* < 0.001) before returning to baseline levels (Figure 4A). There were no significant or meaningful changes in pain interference, sleep disturbance, depression, or anxiety (Figure 4B–E). Global pain showed significant but not meaningful improvement on D30 (*p* = 0.007, Figure 4F). Performance status showed significant but not meaningful worsening on D7 (*p* < 0.001), D14 (*p* < 0.001), D21 (*p* = 0.001), and D30 (*p* = 0.001, Figure 4G). Cognitive function showed significant but not meaningful improvement on D90 (*p* = 0.004, Figure 4H). Changes in social function were not significant or meaningful (Figure 4I). Symptom burden showed significant but not meaningful improvement on D90 (*p* = 0.004, Figure 4J).

### 3.4. Proportions with Clinically Meaningful PRO Scores

**Figure 5:**
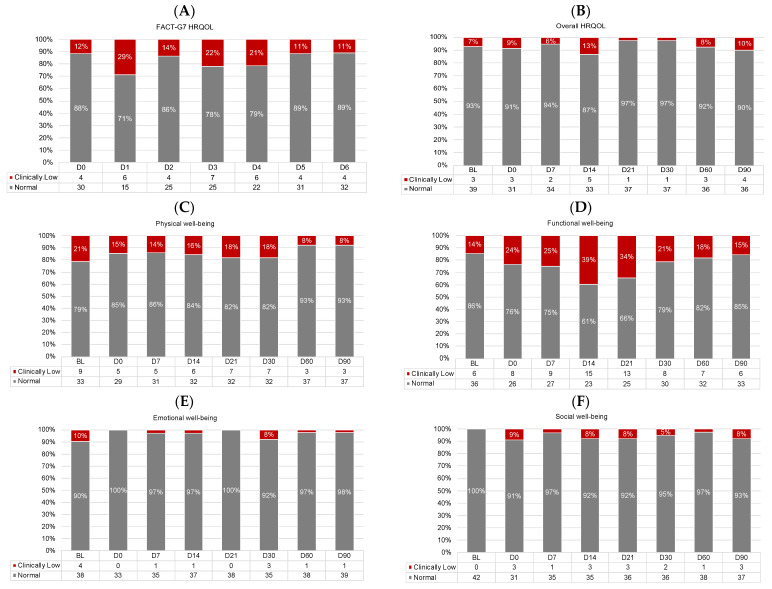
Proportions of participants with normal (gray) and clinically low (red) HRQOL scores at each timepoint for (**A**) daily HRQOL, (**B**) overall HRQOL, (**C**) physical well-being, (**D**) functional well-being, (**E**) emotional well-being, and (**F**) social well-being. Proportions < 5% are not labeled. All frequencies are shown in the legends. D, day. D0 was the day of CAR T-cell infusion.

Figure 5 shows the proportions of participants with normal vs. low HRQOL across timepoints. There were no differences in the proportion of participants with low HRQOL at baseline vs. D90 for any HRQOL outcome (logistic regression *p*-values > 0.01). Functional well-being appeared most impaired, with up to 39% of participants reporting low functional well-being (D14; Figure 5D).

**Figure 6:**
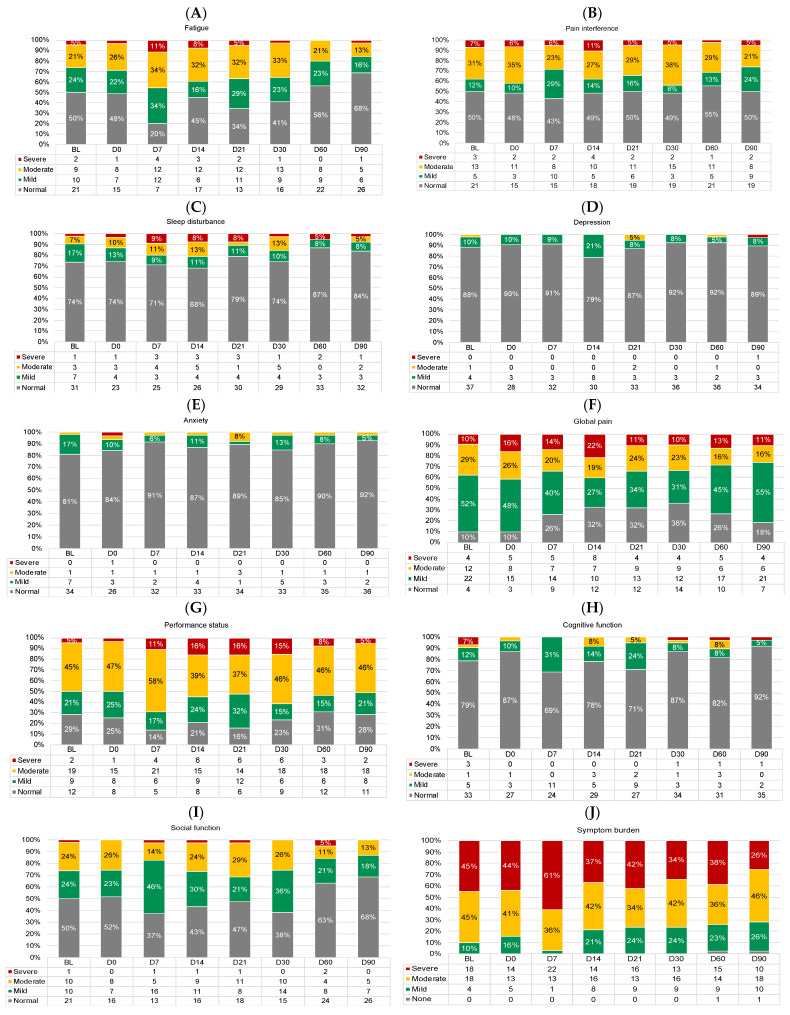
Proportions of participants with normal/none (gray), mild (green), moderate (yellow), and severe (red) symptoms at each timepoint for (**A**) fatigue, (**B**) pain interference, (**C**) sleep disturbance, (**D**) depression, (**E**) anxiety, (**F**) global pain, (**G**) performance status, (**H**) cognitive function, (**I**) social function, and (**J**) overall symptom burden. Proportions < 5% are not labeled. All frequencies are shown in the legend. D, day. D0 was the day of CAR T-cell infusion.

Figure 6 shows the proportions of participants with normal/none, mild, moderate, and severe symptoms across timepoints. There were no differences in the proportion of participants with at least moderate symptoms at baseline vs. D90 for any symptom outcome (logistic regression *p*-values > 0.01). Performance status and overall symptom burden appeared most impaired, with ≥50% of participants reporting at least moderately impaired performance status at all timepoints (Figure 6G) and up to 97% of participants reporting at least moderate overall symptom burden (D7; Figure 6J).

**Figure 7:**
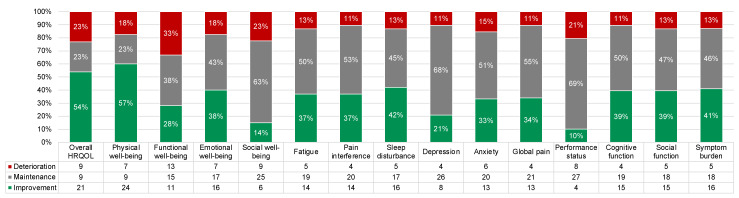
Proportions of participants with clinically meaningful PRO improvement (green), deterioration (red), and maintenance (i.e., no change; gray) from baseline to day 90. All frequencies are shown in the legend.

Figure 7 shows the proportion of participants with clinically meaningful PRO improvement, deterioration, and maintenance from baseline to D90. Overall, most participants had clinically meaningful improvement (range: 10–57%) or maintenance (range: 23–69%). The proportion of participants with meaningful improvement was largest for overall HRQOL (54%) and physical well-being (57%). The proportion with meaningful deterioration was largest for functional well-being (33%).

### 3.5. Time to Stable PRO Change

Median time to stable deterioration was 14 days for functional well-being, and median time to stable improvement was 60 days for physical and emotional well-being (Supplementary Table S5). Median time to stable change was not reached for the other PROs (i.e., <50% of participants had stable change by D90).

## 4. Discussion

This was the first study to evaluate PROs of SOC ide-cel among real-world RRMM patients [18]. In this study, 71% of participants would not have met eligibility criteria for the phase 2 KarMMa trial. We observed significant and meaningful worsening of PROs (i.e., fatigue, functional well-being) as early as D7 post-infusion, which later rebounded to baseline levels. We also observed significant and meaningful improvements in PROs (i.e., overall HRQOL, physical well-being) by D60, which were sustained through D90. Most participants reported clinically meaningful improvement or maintenance of PROs from baseline to D90.

Our results are similar to those of the KarMMa trial but tempered in the context of shorter follow-up. The KarMMa trial found significant and meaningful improvements in most PROs by month 1 or 2 that were sustained over 12 months or more, (e.g., overall HRQOL, physical and cognitive function, pain, fatigue) [14]. Similarly, we found significant, meaningful, and sustained improvements in overall HRQOL and physical well-being by D60 (i.e., month 2) and significant improvements in cognitive function and global pain that did not exceed MID thresholds. In contrast to the KarMMa trial, participants reported worsened fatigue in the week post-infusion before rebounding to baseline levels. Discrepancies between studies could possibly be explained by using different PRO measures. Alternatively, participant cohorts may be demographically and clinically different. For example, real-world RRMM patients in our study were older than KarMMa trial participants (median 66 vs. 61 years), and our study included more patients with ECOG performance status ≥ 2 (10% vs. 2%) and penta-refractory disease (38% vs. 26%) [14]. These differences may reflect characteristics of patients deemed ineligible vs. eligible to participate in clinical trials, and thus, findings from our study are likely more generalizable to the broader real-world population of RRMM patients treated in SOC.

Findings have direct implications for patient education and clinical care, as PROs should be considered throughout the clinical management of RRMM [43]. Clinicians rely on real-world evidence when considering treatment approaches and educating patients, and patient education is a critical component of patient-centered care and informed treatment decision-making [44,45]. Our findings can inform patient education for SOC ide-cel with regard to the potential impacts on key PROs. In addition, findings can be used to identify targets for supportive behavioral and pharmacologic interventions to maximize survivorship outcomes for individual patients post-ide-cel.

Strengths of this study include a rigorous schedule of prospective PRO data collection using validated measures from pre-treatment through D90 post-infusion, which allowed for a nuanced evaluation of how PRO scores changed in the first three months post-ide-cel. Assessment response rates were high, supporting the feasibility of collecting PROs in real-world settings. In addition, we considered both statistical significance and clinical meaningfulness, and we documented a high prevalence of clinically meaningful symptomatology in this population.

Limitations include a relatively small sample size of *n* = 42. This study was limited to a single institution offering commercial ide-cel, which only became available in March 2021. Participants were mostly non-Hispanic White and highly educated, which reflects the characteristics of patients who had access to and received CAR T at an NCI-designated comprehensive cancer center. Studies show that racial and ethnic minority RRMM patients are underrepresented in CAR T clinical trials and are less likely to receive commercial CAR T [46,47]. Thus, future studies should replicate our findings among diverse cohorts that are representative of the broader RRMM population. Finally, follow-up was limited to D90 post-infusion. Studies with longer follow-up are needed to understand PROs further into the trajectory of real-world post-CAR T survivorship.

## 5. Conclusions

This study was the first to investigate the effects of SOC ide-cel on PROs among real-world RRMM patients who largely would not have been eligible for clinical trials. Overall, participants reported significant and meaningful improvements or maintenance of HRQOL and symptom burden up to 90 days post-treatment. The results can be used to inform patient education approaches, treatment decision-making, and early supportive interventions to improve post-CAR T survivorship outcomes among real-world patients with RRMM.

## Figures and Tables

**Table 1:** Baseline sociodemographic and clinical characteristics (*n* = 42).

Characteristics	*n* (%)
Median age, years (range)	66 (43–81)
Sex	
Female	21 (50)
Male	21 (50)
Race/ethnicity	
Non-Hispanic White	29 (69)
Hispanic	6 (14)
Non-Hispanic Black	5 (12)
Non-Hispanic American Indian or Alaska Native	1 (2)
Non-Hispanic Asian	1 (2)
Married	29 (69)
Employment	
Retired	23 (55)
Employed full-time or part-time	9 (21)
Disabled or on leave without pay	9 (21)
Homemaker	1 (2)
Highest education completed	
High school	4 (10)
Partial college or specialized training	14 (33)
College or university	14 (33)
Graduate degree	10 (24)
Annual household income	
<USD 60,000	17 (41)
USD 60,000–USD 100,000	13 (31)
>USD 100,000	7 (17)
Prefer not to report	5 (12)
Median Charlson Comorbidity Index (range)	2 (2–18)
Extramedullary disease	17 (41)
High marrow burden	11 (26)
ECOG performance status 0–1	
0–1	38 (91)
≥2	4 (10)
R-ISS stage	
I	7 (17)
II	29 (69)
III	4 (10)
Unknown	2 (5)
Myeloma subtype	
Intact immunoglobin	25 (60)
Light chain	14 (33)
Non-secretory	3 (7)
High-risk cytogenetic abnormalities	
Any	15 (36)
del(17p)	11 (26)
t(4;14)	2 (5)
t(14;16)	3 (7)
Bridging therapy	22 (52)
Prior therapies *	
Median prior therapies (range)	6 (4–16)
Prior autologous stem cell transplant	36 (86)
Prior anti-BCMA therapy	7 (21)
Refractory status *	
Refractory to immunomodulatory agents	39 (93)
Refractory to proteasome inhibitors	37 (88)
Refractory to anti-CD38 antibodies	39 (93)
Double-refractory	36 (86)
Triple-refractory	34 (81)
Penta-refractory	16 (38)
Median CAR T-cell dose, million cells (range)	413.1 (329.7–457.7)
Met KarMMa eligibility criteria; *n* (%)	12 (29)

Abbreviations: ECOG, Eastern Cooperative Oncology Group; R-ISS, Revised International Staging System. Notes: Percentages may not sum to 100 due to rounding. High marrow burden was defined as ≥50% CD138-positive plasma cells in pre-treatment bone marrow core biopsy. High-risk cytogenetics included del (17p), t (4;14), and t (14;16). Immunomodulatory agents included lenalidomide and pomalidomide. Proteasome inhibitors included bortezomib and carfilzomib. Anti-CD38 antibody monoclonal antibodies included daratumumab and isatuximab. Double-refractory disease was defined as refractory to an immunomodulatory agent and a proteasome inhibitor. Triple-refractory disease was defined as refractory to an immunomodulatory agent, a proteasome inhibitor, and an anti-CD38 monoclonal antibody. Penta-refractory disease was defined as refractory to lenalidomide, pomalidomide, bortezomib, carfilzomib, and daratumumab or isatuximab. * Categories are not mutually exclusive.

**Table 2:** Safety and clinical outcomes in the first 90 days post-CAR T-cell infusion (*n* = 42).

Safety Outcomes	*n* (%)
Hospitalization	
Median hospital stay, days (range)	8 (6–68)
Median intensive care unit stay (*n* = 4), days (range)	6 (4–10)
CRS	
Any	34 (81)
Grade 1	29 (69)
Grade 2	5 (12)
Grade 3	0 (-)
Grade 4	0 (-)
Median time to maximum severity, days (range)	1 (0–13)
Median duration, days (range)	2.5 (1–6)
Neurotoxicity	
Any	4 (10)
Grade 1	2 (5)
Grade 2	0 (-)
Grade 3	1 (2)
Grade 4	1 (2)
Median time to maximum severity, days (range)	3 (1–5)
Median duration, days (range)	1.5 (1–6)
Supportive care for CRS and neurotoxicity *	
Tocilizumab	34 (81)
Corticosteroid	14 (33)
Anakinra	1 (2)
Maximum ferritin, median ng/mL (range)	816 (61–39,188)
Maximum CRP, median mg/dL (range)	5.8 (0.6–32.8)
Any infection	17 (41)
Hematologic toxicity *	
Neutropenia	
Any	41 (100)
Grade 1	2 (5)
Grade 2	6 (15)
Grade ≥ 3	33 (81)
Anemia	
Any	41 (100)
Grade 1	6 (14)
Grade 2	18 (43)
Grade ≥ 3	17 (41)
Thrombocytopenia	
Any	40 (98)
Grade 1	12 (29)
Grade 2	7 (17)
Grade ≥ 3	21 (51)
Supportive care for hematologic toxicity ^†^	
Granulocyte colony stimulating factor	34 (81)
Thrombopoietin agonist	1 (2)
**Clinical Outcomes**	***n*** **(%)**
Best overall response by day 90	
Complete response or better	16 (38)
Very good partial response	2 (5)
Partial response	13 (31)
Stable disease/minor response	5 (12)
Progressive disease	5 (12)
Died or progressed before day 90	1 (2)
Median time to first response, days (range)	29 (27–125)
Median time to complete response or better, days (range)	28 (27–91)
Minimal residual disease negativity at 10^−6^	12 (29)

Abbreviations: CRP, C-reactive protein; CRS, cytokine release syndrome. Notes: Percentages may not sum to 100 due to rounding. * Not applicable for one participant who passed away prior to day 7; thus, percentages were calculated out of *n* = 41. **^†^** Categories are not mutually exclusive.

**Table 3:** Means (standard deviations) of patient-reported outcomes at each timepoint.

	Baseline *n* = 42	D0 *n* = 34	D7 *n* = 36	D14 *n* = 38	D21 *n* = 39	D30 *n* = 39	D60 *n* = 40	D90 *n* = 40
FACT-G								
Overall HRQOL	79.2 (12.4)	78.4 (14.0)	79.3 (9.0)	77.5 (11.6)	77.6 (14.1)	77.8 (15.7)	83.2 (16.7)	83.8 (15.9)
Functional well-being	16.8 (5.8)	14.8 (5.1)	14.8 (4.8)	13.5 (5.3)	14.4 (5.6)	15.2 (4.9)	17.3 (5.8)	18.2 (5.7)
Physical well-being	19.3 (5.3)	20.3 (4.6)	20.0 (5.2)	20.6 (4.6)	20.2 (4.7)	20.7 (5.3)	22.4 (4.5)	22.0 (5.3)
Emotional well-being	19.0 (3.7)	20.4 (2.0)	20.6 (2.7)	20.5 (2.8)	20.7 (2.5)	19.9 (3.6)	20.5 (3.2)	20.5 (3.7)
Social well-being	24.1 (3.5)	23.9 (5.8)	24.0 (3.5)	22.8 (5.2)	23.1 (4.1)	23.5 (4.1)	24.5 (3.2)	23.7 (3.8)
PROMIS-29+2 Profile v2.1								
Fatigue	53.6 (8.6)	54.8 (7.9)	58.8 (8.9)	56.2 (9.5)	56.4 (8.3)	55.7 (8.0)	51.7 (9.9)	51.1 (9.6)
Pain interference	55.0 (10.0)	55.3 (10.2)	54.1 (9.4)	54.7 (11.4)	54.5 (10.5)	53.8 (10.8)	52.3 (9.9)	52.1 (9.7)
Sleep disturbance	50.3 (8.5)	52.3 (8.1)	52.1 (10.5)	50.6 (11.1)	49.3 (10.1)	49.7 (10.3)	47.9 (9.3)	46.7 (10.3)
Depression	46.4 (6.6)	45.4 (5.7)	45.2 (5.9)	46.7 (6.5)	45.9 (7.0)	46.6 (5.9)	45.1 (6.4)	45.2 (7.1)
Anxiety	47.4 (6.4)	47.8 (7.3)	45.4 (6.4)	45.9 (7.0)	45.5 (7.3)	46.4 (7.3)	44.3 (6.4)	45.0 (6.2)
Global pain	3.7 (2.5)	3.9 (2.4)	3.1 (2.7)	3.3 (3.0)	3.0 (2.6)	2.8 (2.6)	3.1 (2.6)	2.9 (2.4)
Performance status	41.9 (9.4)	40.4 (8.6)	37.9 (6.6)	38.5 (7.9)	38.3 (8.8)	38.6 (8.4)	41.7 (10.2)	41.5 (8.5)
Cognitive function	50.7 (9.2)	52.1 (6.7)	51.1 (7.0)	51.3 (7.0)	50.3 (7.9)	52.4 (7.5)	53.1 (9.2)	55.2 (8.0)
Social function	47.0 (10.5)	48.0 (8.9)	46.1 (7.9)	45.3 (7.7)	45.0 (7.7)	45.7 (7.9)	49.2 (10.2)	50.1 (9.5)
Overall symptom burden	3.2 (0.8)	3.1 (0.9)	3.5 (0.6)	3.0 (0.8)	3.0 (0.8)	2.9 (0.8)	2.9 (0.9)	2.8 (0.9)
**Daily Measure**	**D0** ***n* = 34**	**D1** ***n* = 21**	**D2** ***n* = 29**	**D3** ***n* = 32**	**D4** ***n* = 28**	**D5** ***n* = 35**	**D6** ***n* = 36**	
FACT-G7 HRQOL	16.1 (4.6)	16.6 (5.0)	17.1 (3.6)	16.2 (3.7)	16.0 (3.9)	17.1 (3.3)	17.9 (3.8)	

Abbreviations: D, day. Notes: Baseline was completed at a median of 7.5 days before the start of conditioning chemotherapy (range: 37 days before–4 days after) and a median of 12.5 days before D0 (range 1–42 days). D0 was the day of CAR T-cell infusion. For FACT-G, mean scores for overall HRQOL ≤ 70, functional well-being ≤ 14, physical well-being ≤ 18, emotional well-being ≤ 15, and social well-being ≤ 19 indicated clinically low HRQOL. For FACT-G7 daily measure, mean scores ≤ 13 indicated clinically low HRQOL. For PROMIS fatigue, pain interference, sleep disturbance, depression, and anxiety, mean scores of 55–59 were mild, 60–69 were moderate, and ≥70 were severe. For PROMIS global pain, scores of 1–4 were mild, 5–6 were moderate, and ≥7 were severe. For PROMIS physical, cognitive, and social function, scores ≤ 30 were severe, 31–40 were moderate, and 41–45 were mild. For overall symptom burden, scores of 1–1.99 were mild, 2–2.99 were moderate, and ≥3 were severe.

## Data Availability

Data are available from the corresponding author upon reasonable request. The data are not publicly available due to privacy restrictions.

## Supplementary Materials

The following supporting information can be downloaded at: https://www.mdpi.com/article/10.3390/cancers15194711/s1, Table S1. Schedule and content of patient-reported outcome assessments. Table S2. Associations between patient characteristics and patient-reported outcomes at baseline (N = 42). Table S3. Results of linear mixed models evaluating estimated mean change from baseline for the HRQOL outcomes. Table S4. Results of linear mixed models evaluating estimated mean changes from baseline for the symptom outcomes. Table S5. Proportions of participants with and median time to stable improvement and deterioration for each patient-reported outcome.
