# Supplementary material for: Patient-Reported Outcomes among Multiple Myeloma Patients Treated with Standard of Care Idecabtagene Vicleucel
Source: Cancers (Basel). 2023 Sep 25;15(19):4711. doi: 10.3390/cancers15194711 (PMC10571575; doi:10.3390/cancers15194711)
Supplement: Supplementary file 1 [file cancers-15-04711-s001.zip › cancers-2603780-supplementary.pdf]

Supplemental Table S1. Schedule and content of patient-reported outcome assessments.

Measure	Construct	BL	D0	D1-D6	D7	D14	D21	D30	D60	D90
Demographics Survey	Demographics	X								
CCI	Comorbidities	X								
FACT-G	HRQOL	X	X		X	X	X	X	X	X
FACT-G7	HRQOL		X*	X						
PROMIS-29+2 Profile v2.1	Symptoms	X	X		X	X	X	X	X	X
PRO-CTCAE items	Symptoms	X	X		X	X	X	X	X	X

Abbreviations: BL, baseline; CCI, Charlson Comorbidity Index; D, day; FACT-G, Functional Assessment of Cancer Therapy-General; HRQOL, health-related quality of life; PRO-CTCAE, Patient-Reported Outcomes Version of the Common Terminology Criteria for Adverse Events; PROMIS, Patient-Reported Outcomes Measurement Information System. Notes: D0 was day of CAR T-cell infusion. *A FACT-G7 score was derived from the FACT-G on D0 to facilitate statistical analyses.

Supplemental Table S2. Associations between patient characteristics and patient-reported outcomes at baseline (N=42).

	**Age**		**Sex**		**Race/ Ethnicity**		**Education**		**Extra-medullary disease**		**High marrow burden**		**Bridging therapy**		**No. of prior therapies**		**Refractory status**		**KarMMa eligibility**	
	t	*p*	*t*	*p*	*t*	*p*	*t*	*p*	*t*	*p*	*t*	*p*	*t*	*p*	*t*	*p*	*t*	*p*	*t*	*p*
FACT-G																				
Overall HRQOL	-0.33	0.74	1.05	0.30	0.85	0.40	1.75	0.09	-1.01	0.32	-0.79	0.43	-1.77	0.08	-0.33	0.75	0.25	0.80	-0.23	0.82
Functional well-being	-0.59	0.56	0.60	0.55	0.68	0.50	1.67	0.10	-0.73	0.47	-0.33	0.74	-1.60	0.12	0.13	0.90	0.03	0.98	-0.20	0.85
Physical well-being	0.50	0.62	0.45	0.66	1.82	0.08	0.89	0.38	-2.78	0.008*	-0.86	0.40	-1.96	0.06	0.44	0.67	-1.73	0.09	-0.66	0.51
Emotional well-being	-0.54	0.59	0.21	0.84	0.21	0.84	0.88	0.38	1.35	0.19	-1.69	0.10	-0.63	0.53	-2.19	0.03	118	0.24	0.07	0.95
Social well-being	-0.41	0.68	1.85	0.07	-1.00	0.32	4.56	<0.001*	0.13	0.90	0.76	0.45	0.03	0.98	0.14	0.89	2.25	0.03	0.45	0.66
PROMIS-29+2 Profile v2.1																				
Fatigue	1.23	0.23	-1.18	0.24	1.32	0.20	-0.25	0.80	1.29	0.21	-0.18	0.86	2.35	0.02	-0.36	0.72	1.20	0.24	0.70	0.49
Pain interference	-0.30	0.76	-2.04	0.05	-1.08	0.29	-0.55	0.58	2.19	0.03	0.32	0.75	0.47	0.64	-0.56	0.58	0.78	0.44	2.49	0.02
Sleep disturbance	-1.29	0.20	-0.88	0.39	-0.55	0.58	-0.14	0.89	1.81	0.08	1.21	0.23	0.07	0.94	1.50	0.14	1.59	0.12	1.57	0.12
Depression	1.02	0.31	-1.87	0.07	0.84	0.41	-2.30	0.03	0.05	0.96	0.03	0.97	1.54	0.13	0.66	0.51	-0.53	0.60	0.41	0.69
Anxiety	0.43	0.67	-0.98	0.33	0.34	0.74	-0.02	0.99	-0.73	0.49	0.95	0.35	1.04	0.30	0.80	0.43	-0.32	0.75	-0.55	0.58
Global pain	-0.77	0.45	-1.25	0.22	-0.90	0.37	-0.14	0.89	4.18	<0.001*	0.16	0.87	0.28	0.78	-0.31	0.76	0.71	0.48	2.06	0.05
Performance status	1.80	0.08	0.42	0.68	1.96	0.06	0.00	>0.99	-3.33	0.002*	-0.60	0.55	-1.03	0.31	-0.12	0.91	-1.88	0.07	-1.35	0.19
Cognitive function	-0.22	0.83	-0.13	0.90	0.84	0.40	1.11	0.28	0.37	0.72	1.11	0.28	-1.17	0.25	0.86	0.40	1.01	0.32	-0.08	0.94
Social function	0.10	0.92	0.20	0.84	0.80	0.43	0.75	0.46	-1.25	0.22	-1.42	0.16	-2.07	0.04	-0.43	0.67	-0.69	0.50	-0.40	0.69
Overall symptom burden	-1.06	0.29	0.02	0.98	-0.72	0.48	-0.85	0.40	4.27	<0.001*	-0.40	0.69	1.20	0.24	-0.46	0.65	2.43	0.02	1.60	0.12

Abbreviations: FACT-G, Functional Assessment of Cancer Therapy-General; HRQOL, health-related quality of life; PROMIS, Patient-Reported Outcomes Measurement Information System. Notes: T-values and p-values represent results of independent samples t-tests assessing differences in patient-reported outcome scores at baseline by patient characteristics. High marrow burden was defined as ≥50% CD138-positive plasma cells in pre-treatment bone marrow core biopsy. Patient characteristics were coded as follows: Sex: 1=female vs. 0=male; Race/ethnicity: 1=non-Hispanic White vs. 0=other; Education: 1=college degree or more vs. 0=less than college degree; Extramedullary disease: 1=yes vs. 0=no; High marrow burden: 1=yes vs. 0=no; Bridging therapy: 1=yes vs. 0=no; Refractory status: 1=penta-refractory vs. 0=not penta-refractory; KarMMa eligibility: 1=met vs. 0=did not meet. *p<0.01.

Supplemental Table S3. Results of linear mixed models evaluating estimated mean change from baseline for the HRQOL outcomes.

	**D0**	**D7**	**D14**	**D21**	**D30**	**D60**	**D90**
Overall HRQOL							
Estimate	0.11	-1.33	-2.42	-1.53	-0.65	4.80†	5.01†
Standard error	1.90	1.85	1.82	1.82	1.82	1.80	1.80
t-value	0.06	-0.72	-1.33	-0.84	-0.36	2.66	2.78
p-value	0.95	0.47	0.19	0.40	0.72	0.008*	0.006*
Physical well-being							
Estimate	0.88	0.17	1.00	0.76	1.14	2.82†	2.40†
Standard error	0.79	0.78	0.77	0.76	0.76	0.76	0.76
t-value	1.10	0.22	1.30	0.99	1.49	3.73	3.17
p-value	0.27	0.83	0.20	0.32	0.14	<0.001*	0.002*
Functional well-being							
Estimate	-1.83	-2.49‡	-3.45‡	-2.68‡	-1.76	0.27	1.05
Standard error	0.86	0.84	0.82	0.82	0.82	0.82	0.82
t-value	-2.13	-2.97	-4.19	-3.25	-2.13	0.33	1.29
p-value	0.03	0.003*	<0.001*	0.001*	0.03	0.75	0.20
Emotional well-being							
Estimate	1.01	1.27	1.28	1.32	0.61	1.29	1.20
Standard error	0.50	0.49	0.48	0.48	0.48	0.48	0.48
t-value	2.01	2.59	2.65	2.73	1.27	2.69	2.51
p-value	0.046	0.01	0.009*	0.007*	0.21	0.008*	0.01
Social well-being							
Estimate	0.04	-0.25	-1.21	-1.11	-0.78	0.32	-0.40
Standard error	0.68	0.67	0.65	0.65	0.66	0.65	0.64
t-value	0.06	-0.37	-1.85	-1.71	-1.19	0.50	-0.61
p-value	0.96	0.71	0.07	0.09	0.24	0.62	0.54
**Daily Measure**	**D0**	**D1**	**D2**	**D3**	**D4**	**D5**	**D6**
FACT-G7 HRQOL							
Estimate	-0.14	0.52	1.13	0.51	-0.05	1.08	2.00†
Standard error	0.77	0.89	0.80	0.77	0.81	0.75	0.75
t-value	-0.18	0.58	1.42	0.66	-0.06	1.43	2.67
p-value	0.86	0.56	0.16	0.51	0.95	0.15	0.008*

Abbreviations: D, day; FACT-G, Functional Assessment of Cancer Therapy-General; HRQOL, health-related quality of life. Notes: D0 was day of CAR T-cell infusion. T-values and p-values represent results of linear mixed models assessing change from baseline. *p<0.01. †Clinically meaningful improvement. ‡Clinically meaningful deterioration.

Supplemental Table S4. Results of linear mixed models evaluating estimated mean changes from baseline for the symptom outcomes.

	D0	D7	D14	D21	D30	D60	D90
Fatigue							
Estimate	0.95	5.07‡	2.69	2.96	2.37	-1.60	-2.04
Standard error	1.30	1.25	1.22	1.22	1.21	1.21	1.22
t-value	0.73	4.05	2.20	2.43	1.95	-1.32	-1.67
p-value	0.46	<0.001*	0.03	0.02	0.05	0.19	0.10
Pain interference							
Estimate	0.06	-0.71	-0.19	-0.81	-0.79	-2.18	-2.37
Standard error	1.29	1.24	1.22	1.21	1.20	1.21	1.21
t-value	0.05	-0.57	-0.16	-0.67	-0.66	-1.81	-1.96
p-value	0.96	0.57	0.87	0.51	0.51	0.07	0.05
Sleep disturbance							
Estimate	1.70	1.84	0.38	-0.97	-0.35	-1.93	-3.07
Standard error	1.49	1.44	1.40	1.40	1.39	1.40	1.40
t-value	1.14	1.28	0.27	-0.69	-0.25	-1.38	-2.19
p-value	0.26	0.20	0.79	0.49	0.80	0.17	0.03
Depression							
Estimate	-0.41	-1.10	0.29	-0.24	0.46	-1.08	-0.73
Standard error	1.03	0.99	0.96	0.96	0.96	0.96	0.96
t-value	-0.40	-1.11	0.30	-0.25	0.48	-1.13	-0.76
p-value	0.69	0.27	0.76	0.80	0.63	0.26	0.45
Anxiety							
Estimate	1.24	-1.53	-1.39	-1.49	-0.56	-2.69	-1.90
Standard error	1.12	1.08	1.05	1.05	1.05	1.05	1.05
t-value	1.11	-1.42	-1.32	-1.41	-0.53	-2.57	-1.82
p-value	0.270	0.16	0.19	0.16	0.60	0.01	0.07
Global pain							
Estimate	0.07	-0.58	-0.37	-0.71	-0.84	-0.49	-0.73
Standard error	0.33	0.32	0.31	0.31	0.31	0.31	0.31
t-value	0.21	-1.82	-1.19	-2.29	-2.73	-1.58	-2.34
p-value	0.84	0.07	0.24	0.02	0.007*	0.12	0.02
Performance status							
Estimate	-1.87	-4.49	-3.90	-3.48	-3.45	-0.45	-0.57
Standard error	1.04	1.00	0.98	0.98	0.98	0.98	0.98
t-value	-1.80	-4.49	-3.96	-3.54	-3.54	-0.46	-0.58
p-value	0.07	<0.001*	<0.001*	0.001*	0.001*	0.65	0.56
Cognitive function							
Estimate	1.33	-0.03	0.58	-0.28	1.48	1.88	3.98
Standard error	1.47	1.42	1.39	1.38	1.37	1.38	1.38
t-value	0.90	-0.02	0.41	-0.20	1.08	1.36	2.88
p-value	0.37	0.99	0.68	0.84	0.28	0.18	0.004*
Social function							
Estimate	1.13	-1.59	-2.44	-2.49	-1.56	1.77	2.69
Standard error	1.25	1.21	1.19	1.18	1.17	1.18	1.18
t-value	0.90	-1.32	-2.05	-2.12	-1.34	1.51	2.28
p-value	0.37	0.19	0.041	0.04	0.18	0.13	0.02
Overall symptom burden							
Estimate	-0.04	0.34	-0.15	-0.13	-0.25	-0.23	-0.39
Standard error	0.14	0.14	0.13	0.13	0.13	0.13	0.13
t-value	-0.26	2.48	-1.13	-0.95	-1.84	-1.71	-2.95
p-value	0.80	0.01	0.26	0.34	0.07	0.09	0.004*

Abbreviations: D, day. Notes: D0 was day of CAR T-cell infusion. T-values and p-values represent results of linear mixed models assessing change from baseline. *p<0.01. †Clinically meaningful improvement. ‡Clinically meaningful deterioration.

Supplemental Table S5. Proportions of participants with and median time to stable improvement and deterioration for each patient-reported outcome.

	Stable Improvement		Stable Deterioration	
	**n (%) with stable improvement**	**Median days to stable improvement**	**n (%) with stable deterioration**	**Median days to stable deterioration**
FACT-G				
Overall HRQOL	19 (46.3)	NR	18 (43.9)	NR
Physical well-being	24 (58.5)	60	13 (31.7)	NR
Functional well-being	9 (22.0)	NR	28 (68.3)	14
Emotional well-being	22 (53.7)	60	12 (29.3)	NR
Social well-being	10 (24.4)	NR	11 (26.8)	NR
PROMIS-29 + 2 Profile v2.1				
Fatigue	11 (26.8)	NR	16 (39.0)	NR
Pain interference	16 (39.0)	NR	8 (19.5)	NR
Sleep disturbance	14 (34.1)	NR	10 (24.4)	NR
Depression	8 (19.5)	NR	6 (14.6)	NR
Anxiety	14 (34.1)	NR	7 (17.1)	NR
Global pain	14 (34.1)	NR	5 (12.2)	NR
Performance status	3 (7.3)	NR	16 (39.0)	NR
Cognitive function	14 (34.1)	NR	16 (39.0)	NR
Social function	12 (29.3)	NR	13 (31.7)	NR
Overall symptom burden	17 (41.5)	NR	8 (19.5)	NR

Abbreviations: FACT-G, Functional Assessment of Cancer Therapy-General; HRQOL, health-related quality of life; NR, not reached (i.e., less than half of participants reached stable improvement or deterioration by day 90); PROMIS, Patient-Reported Outcomes Measurement Information System.
